# Mutant *TP53* G245C and R273H promote cellular malignancy in esophageal squamous cell carcinoma

**DOI:** 10.1186/s12860-018-0167-y

**Published:** 2018-08-20

**Authors:** Nan Kang, Yu Wang, Shichao Guo, Yunwei Ou, Guangchao Wang, Jie Chen, Dan Li, Qimin Zhan

**Affiliations:** 1 State Key Laboratory of Molecular Oncology, Chinese Academy of Medical Sciences and Peking Union Medical College, National Cancer Center/Cancer Hospital, Beijing, 100021 China; 2 Department of Pathology, Peking University People’s Hospital, Beijing, 100044 China; 3 Laboratory of Molecular Oncology, Peking University Cancer Hospital and Institute, Beijing, China

**Keywords:** Esophageal squamous cell carcinoma, *TP53* mutation, Cell malignancy, Migration, Invasion, Apoptosis, Cell cycle arrest, Downstream gene

## Abstract

**Background:**

*TP53* gene mutations occur in more than 50% of human cancers and the vast majority of these mutations in human cancers are missense mutations, which broadly occur in DNA binding domain (DBD) (Amino acids 102–292) and mainly reside in six “hotspot” residues. *TP53* G245C and R273H point mutations are two of the most frequent mutations in tumors and have been verified in several different cancers. In the previous study of the whole genome sequencing (WGS), we found some mutations of *TP53* DBD in esophageal squamous cell carcinoma (ESCC) clinical samples. We focused on two high-frequent mutations *TP53* p.G245C and *TP53* p.R273H and investigated their oncogenic roles in ESCC cell lines, p53-defective cell lines H1299 and HCT116 p53−/−.

**Results:**

MTS and colony formation assays showed that mutant *TP53* G245C and R273H increased cell vitality and proliferation. Flow cytometry results revealed inhibition of ultraviolet radiation (UV)- and ionizing radiation (IR)- induced apoptosis and disruption of *TP53*-mediated cell cycle arrest after UV, IR and Nocodazole treatment. Transwell assays indicated that mutant *TP53* G245C and R273H enhanced cell migration and invasion abilities. Moreover, western blot revealed that they were able to suppress the expression of *TP53* downstream genes in the process of apoptosis and cell cycle arrest induced by UV, which suggests that these two mutations can influence apoptosis and growth arrest might be due, at least in part, to down-regulate the expression of P21, GADD45α and PARP.

**Conclusions:**

These results indicate that mutant *TP53* G245C and R273H can lead to more aggressive phenotypes and enhance cancer cell malignancy, which further uncover *TP53* function in carcinogenesis and might be useful in clinical diagnosis and therapy of *TP53* mutant cancers.

**Electronic supplementary material:**

The online version of this article (10.1186/s12860-018-0167-y) contains supplementary material, which is available to authorized users.

## Background

*TP53*, as an important tumor suppressor gene, plays a crucial role in the process of maintenance of normal cellular function. Wild-type (WT) *TP53* can be activated to regulate many cellular programs like cell cycle arrest, DNA repair, apoptosis, autophagy, senescence, metabolic remodeling and innate immunity [1–3]. *TP53* gene mutations occur in more than 50% of human cancers, including liver cancer, breast cancer, bladder cancer, stomach cancer, colon cancer, prostate cancer, soft tissue sarcoma, ovarian cancer, brain tumor, esophageal cancer, lung cancer and osteosarcoma [4, 5]. The vast majority of *TP53* mutations in human cancers are missense mutations, which broadly occur in DBD (Amino acids 102–292) and mainly reside in six “hotspot” residues (p.R175, p.G245, p.R248, p.R249, p.R273, and p.R282) [4, 6, 7].

The majority of *TP53* gene mutations in human cancers abolish its tumor-suppressive function to bind to specific DNA sequences recognized by wild-type *TP53*, which is called loss of function (LOF) [4, 8]. Some *TP53* mutations reduce the reaction with wild-type *TP53* downstream genes, resulting in the inactivation of wild-type *TP53* or its response elements, which lead to gain of oncogenic function (GOF) [9–12]. Moreover, the mutant P53 proteins frequently exhibit a dominant negative effect on the wild-type *TP53* allele by interacting with wild-type *TP53* and reducing cellular concentration of functional wild-type *TP53*, which can form wild-type *TP53* tetramer structure but lose the activity of wild-type *TP53* [1, 3, 4, 13].

As previously reported, *TP53* G245C and R273H point mutations are two of the most frequent mutations in tumors and have been verified in several different cancers [7]. It has been reported that R273H can enhance invasion of lung cancer cells [14] and promote invasion and migration in endometrial cells [8]. G245C has been confirmed to result in changes in the conformation of the *TP53* DNA-binding domain, compared with wild-type *TP53* [15]. However, the properties of such mutations are not well characterized and there is little information on G245C and R273H mutations in ESCC and p53-defective cancer cells. From the previous results of WGS in ESCC patients’ samples [16], we focused on these two mutations and verified their tumorigenicity in ESCC cell lines, p53-defective cell lines H1299 and HCT116 p53−/−. We applied to determine the influence of G245C and R273H mutations of *TP53* on cell proliferation, apoptosis and cell cycle arrest induced by UV, IR and Nocodazole in human cancer cells.

The current study aims to explore the function and impact of *TP53* G245C and R273H mutations on cancer cell proliferation, migration, invasion, apoptosis and cell cycle arrest after UV, IR and Nocodazole treatments, which might serve as a potential diagnostic and therapeutic target in *TP53* mutant cancers.

## Results

### *TP53* G245C and R273H mutations analysis in ESCC patients’ samples and cell lines

**Fig. 1:**
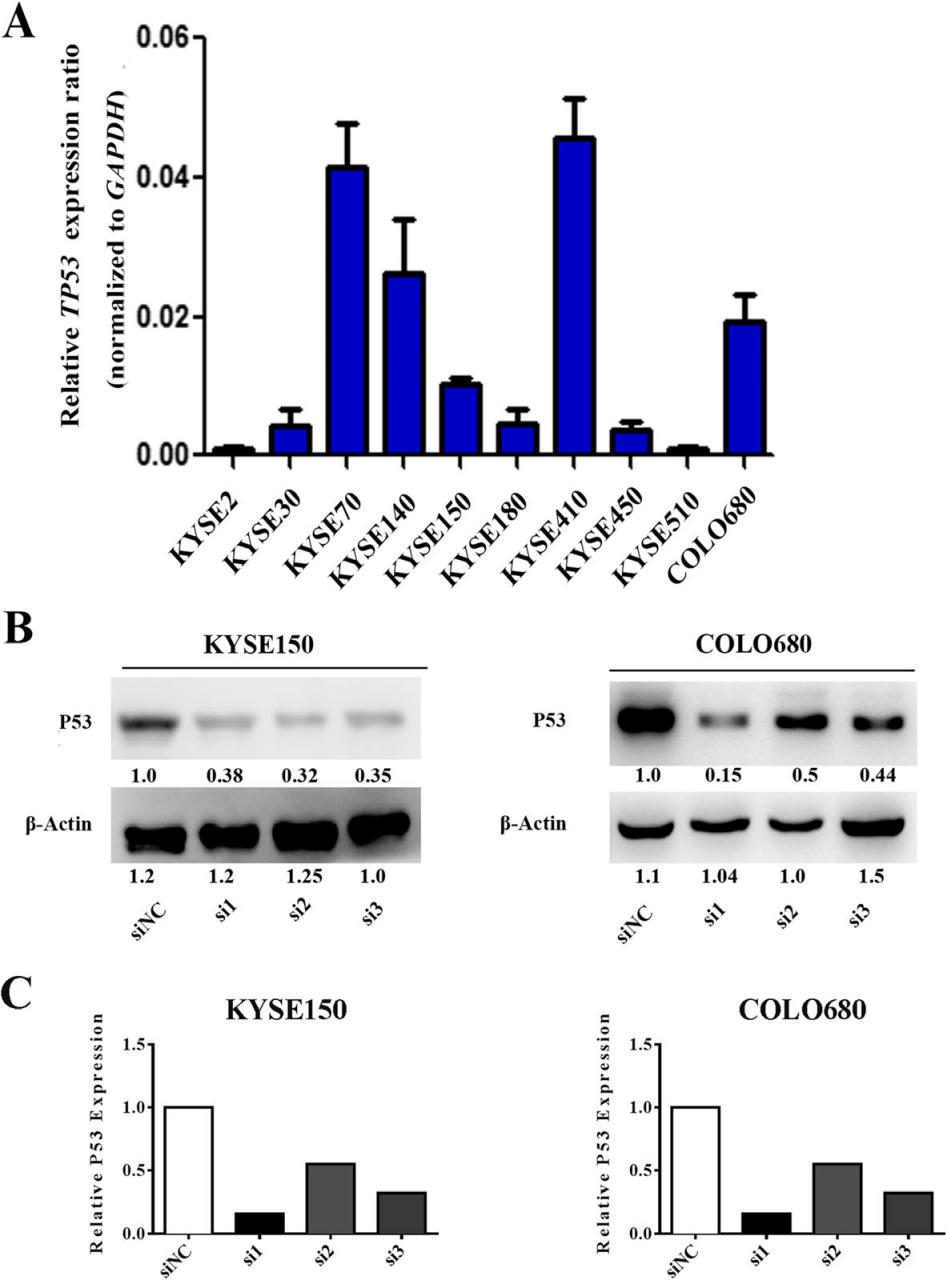
*TP53* expression levels in ESCC cell lines. **a** qRT-PCR analysis of the relative *TP53* mRNA levels in nine ESCC cell lines. *TP53* mRNA levels in KYSE150 and COLO680 were considerate high. **b** Western blot assay was applied to verify the expression of P53 in KYSE150 and COLO680 after siRNAs treatment, which express relatively higher levels of P53 and contain wild-type P53. **c** Gray scale scanning analysis was employed to examine the efficiency of silencing of p53 in KYSE150 and COLO680

According to the previous results of whole genome sequencing (WGS) in ESCC patients’ samples [16], we found that *TP53* somatic mutations were present in sequenced tumors. *TP53* at codons G245 and R273 were identified in respectively as shown in Tables 1 and 2. Total genomic DNA of ten ESCC cell lines were extracted and the mutation sites in 11 *TP53* exons were validated by Sanger Sequencing (Table 3) and their expression were assessed by real time PCR assays (Fig. 1a). Short interfering RNAs (siRNAs) were applied to knock down P53 expression levels in KYSE150 and COLO680 cells, which express wild-type and relatively higher levels of P53. The knockdown efficiency of P53 by siRNAs were verified by Western blot (Fig. 1b). We designed gray scale scanning in KYSE150 and COLO680 to examine the efficiency of silencing of p53, and the result indicated that p53 was knockdown by siRNAs obviously (Fig. 1c). Additionally, siRNAs and *TP53* plasmids were transiently cotransfected in KYSE150 and COLO680 cells, and we validated the effects of *TP53* G245C and R273H mutant transfectants on cellular malignant phenotypes including cell growth, colony formation, migration and invasion.

**Table 1:** *TP53* mutation types in 88 ESCC cases of WGS

*TP53* mutant type	*n*
Missense mutation	53
733G>T(G245C)	1
818G> A(R273H)	4
Nonsense mutation	17
Deletion	7
Splicing region	3

**Table 2:** *TP53* mutation sites in 88 WGS ESCC samples

Sample	Mutant type	CDS mutation	AA mutation
ESCC-010 T	Missense	c.818G > A	p.R273H
ESCC-112 T	Missense	c.818G > A	p.R273H
ESCC-134 T	Missense	c.818G > A	p.R273H
ESCC-147 T	Missense	c.818G > A	p.R273H
ESCC-009 T	Missense	c.733G > T	p.G245C

**Table 3:** Sequenced and assessed *TP53* mutations in Ten ESCC cell lines. Only KYSE150 and COLO680 express the WT *TP53*

Cell line	Codon	Exon	Base variation
KYSE2			
1	72	4	C > G
2	221	6	G > T
KYSE30			
1	72	4	C > G
KYSE70			
1	120	4	G > T
KYSE140			
1	72	4	C > G
2	160	6	A > G
KYSE150			
none	none	none	none
KYSE180			
1	72	4	C > G
2	162	6	T > C
KYSE410			
1	72	4	C > G
2	337	10	C > T
KYSE450			
1	72	4	C > G
2	179	6	A > G
3	339	10	G > T
KYSE510			
1	343	10	G > T
COLO680			
none	none	none	none

### *TP53* G245C and R273H mutants positively modulate tumor cell proliferation activities

**Fig. 2:**
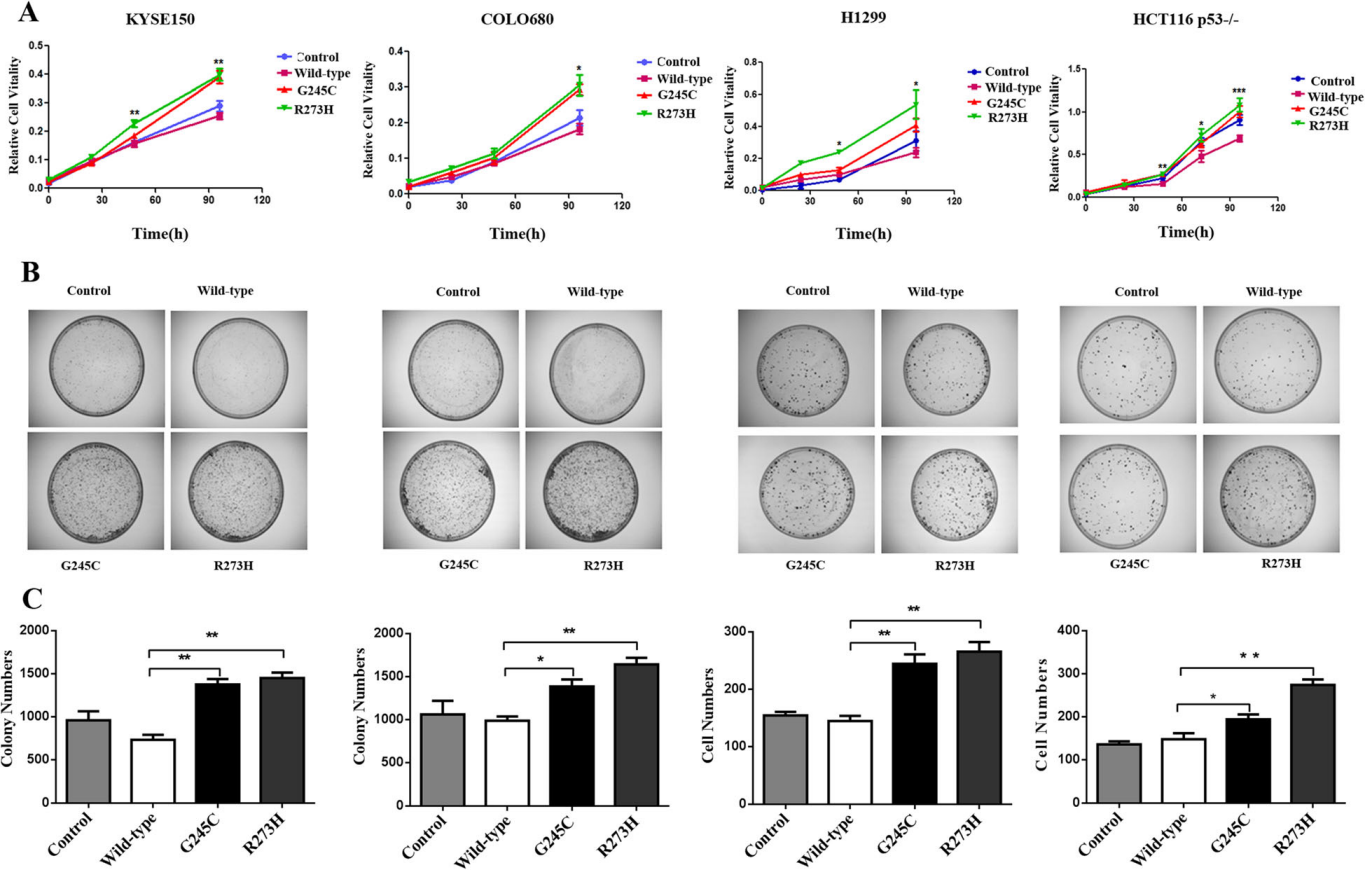
*TP53* G245C and R273H mutants accelerated the proliferation activities of tumor cells. **a** Cell proliferation activity was examined by MTS assay and each cell clusters were counted at 0 h, 24 h, 48 h, 72 h and 96 h. Data was shown as the average from at least three independent experiments. **b** The long-term cell growth activity was tested by colony formation assay in above four tumor cell lines. Cell numbers were counted after 14 days incubation. (*P < 0.05, **P < 0.01, ***P < 0.001)

To determine the role of mutant *TP53* on tumor cell malignancy, MTS assays were used to test the impact of mutant *TP53* on cell proliferation, the different characteristics of the various *TP53* transiently cotransfected cell lines were analyzed and shown in Fig. 2a. The effects of the *TP53* mutations on proliferation were reflected in colony formation assays (Fig. 2b), more colonies were presented in cells expressing mutant *TP53* than that seen in wild-type *TP53* cells. Additionally, the mutant *TP53* led to a significantly greater promotion of cell growth than wild-type *TP53* transfectants. Compared with control cells, wild-type *TP53* suppressed cell proliferation. Taken together, these results illustrated that the *TP53* G245C and R273H mutant cells strengthened cell proliferation abilities.

### *TP53* G245C and R273H mutations positively regulate tumor invasion and migration activities

**Fig. 3:**
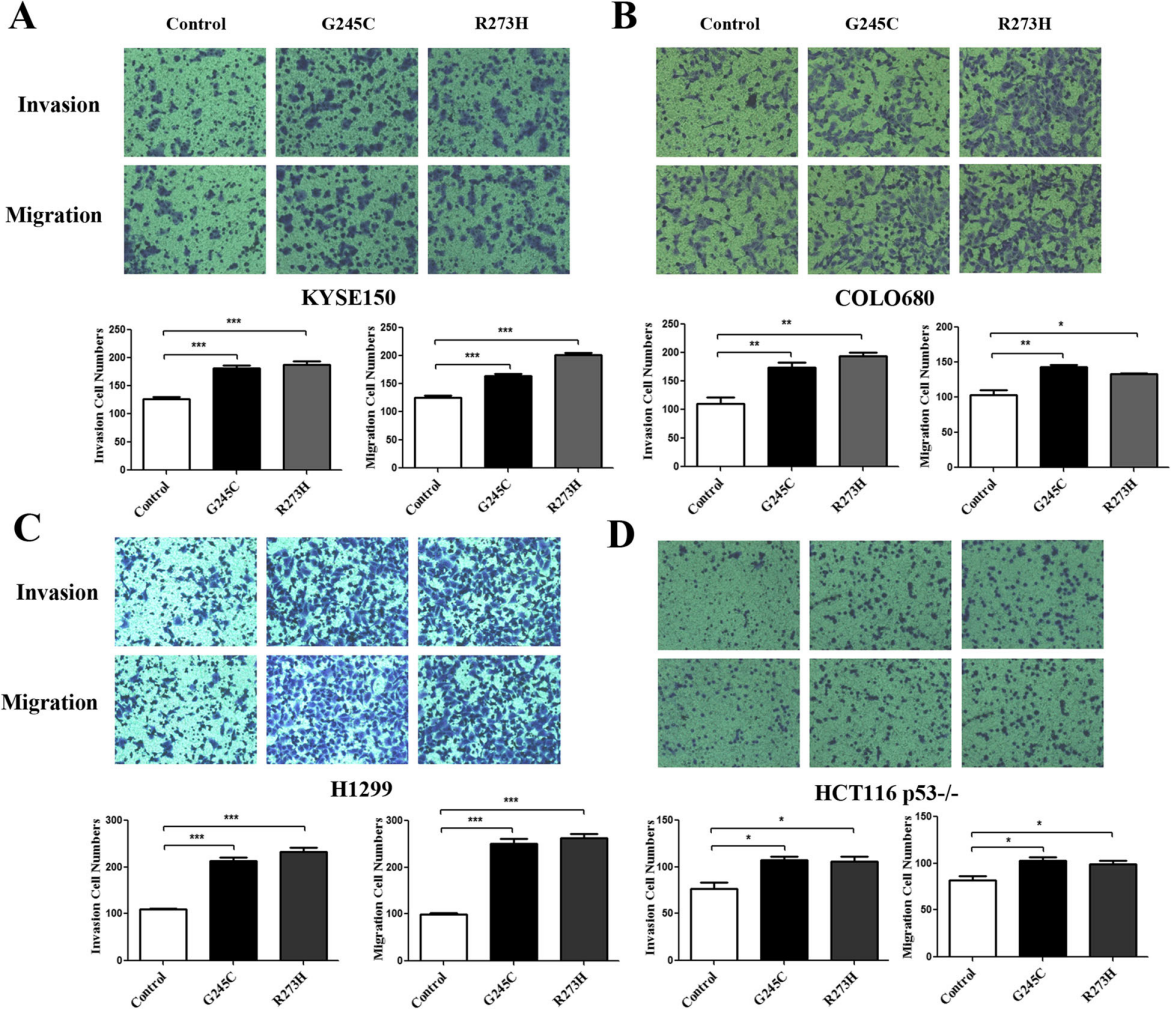
*TP53* G245C and R273H mutants promoted tumor cells invasion and migration. **a** Invasion and migration activities were examined by Matrigel™ invasion chamber in KYSE150 cell line. **b** Invasion and migration activities were assessed in COLO680 cell line. **c** Invasion and migration abilities were verified in H1299 cell line. **d** Invasion and migration activities were tested in HCT116 p53−/− cell line. Data was shown as mean ± SE. (*P < 0.05, **P < 0.01, ***P < 0.001)

We next determined the influence of *TP53* G245C and R273H on invasion and migration activities. Matrigel™ invasion chamber system was employed to test the properties of invasion and migration, the mutant transfectants showed higher levels of membrane penetration abilities. Invasion and migration were significantly higher in cells expressing *TP53* G245C and R273H than vector controls in KYSE150 (Fig. 3a) and COLO680 (Fig. 3b), p53-defective cell lines H1299 (Fig. 3c) and HCT116 p53−/− (Fig. 3d) transfected with relative plasmids. Based on the proliferation and invasion assays, we concluded that *TP53* G245C and R273H mutations are gain of oncogenic function in ESCC cell lines and p53-defective cell lines H1299, HCT116 p53−/− cells.

### The influence of *TP53* G245C and R273H mutants on tumor cell apoptosis and cell cycle arrest

**Fig. 4:**
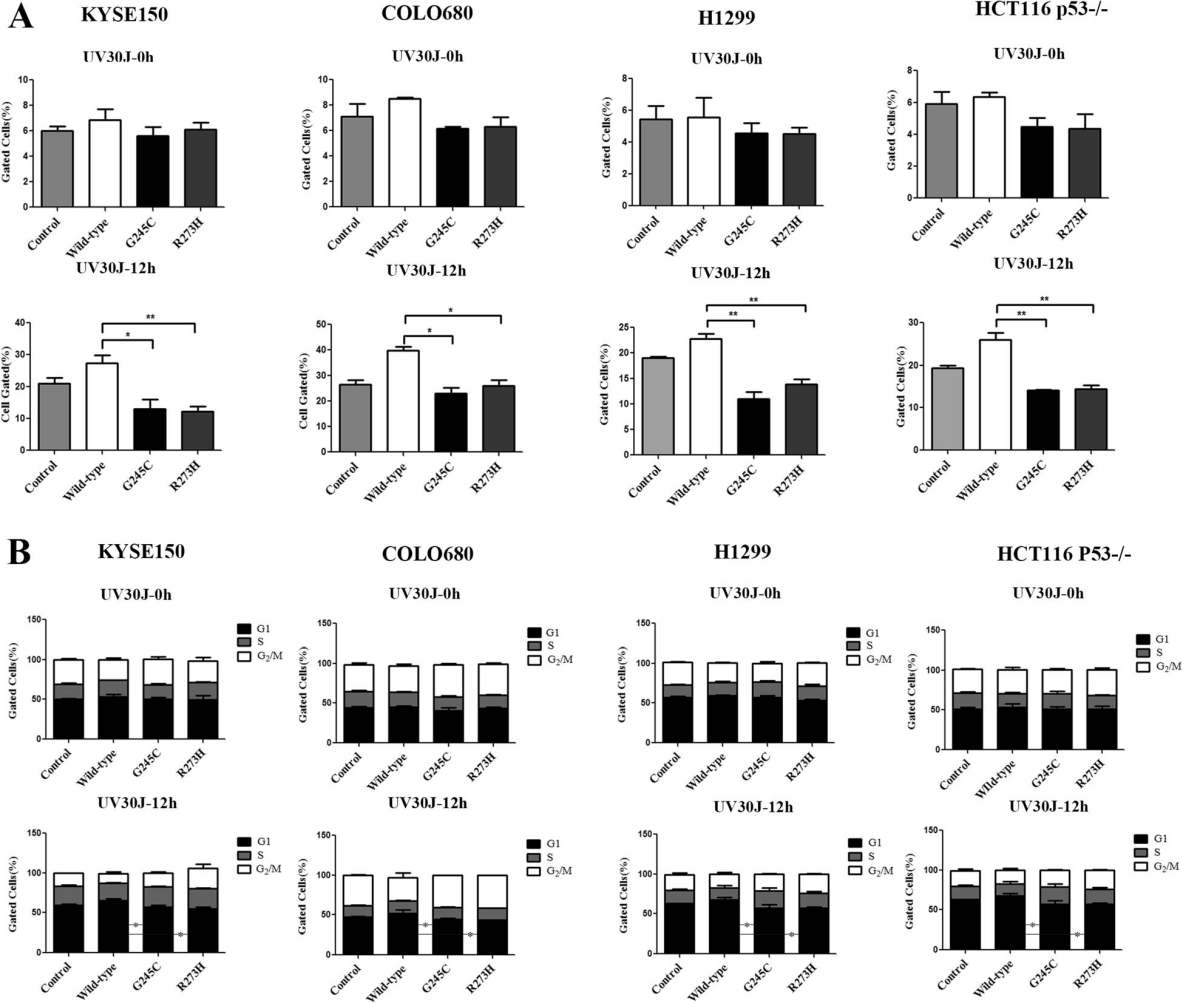
G245C and R273H conferred cells stronger resistance to UV-induced apoptosis and attenuated cell cycle arrest. **a** Apoptosis degree induced by UV 30 J for 0 h and 12 h in cell lines. The data were presented as the portion of cells undergoing apoptosis tested by flow cytometry, the cells were dyed by PI and AnnexinV. **b** Quantitation of cell cycle arrest ratio in cells transfected by WT and mutant *TP53* plasmids after UV 30 J for 0 h and 12 h treatment. Data was shown as the percentage of DNA amounts dyed by PI which was detected by flow cytometry. Data was shown as mean ± SE. (*P < 0.05, **P < 0.01, ***P < 0.001)

We also examined effects of *TP53* G245C and R273H on cell apoptosis and cell cycle arrest following UV, IR and Nocodazole treatments. We assessed UV- and IR-induced apoptosis in KYSE150 and COLO680 cotransfected with control, wild-type *TP53*, *TP53* G245C, *TP53* R273H plasmids and siRNAs as well as H1299, HCT116 p53−/− cells. As shown in Fig. 4a, the percentage of cells undergoing apoptosis after UV 30 J for 12 h treatment was less in G245C and R273H than WT and control cells, compared to UV 30 J for 0 h in ESCC cell lines. Furthermore, the ratio of apoptosis cells after IR 4Gy for 12 h in G245C and R273H was dramatically less than WT and control cells in KYSE150 and COLO680 cell lines (Additional file 1: Figure S1A), which were consistent with the data described above. In the analysis of cell cycle, we found that the abilities of cells performing G1 arrest after UV 30 J for 12 h treatments in the G245C and R273H were weaker than WT and control cells, compared to UV 30 J for 0 h (Fig. 4b). In addition, this phenomenon was also observed in KYSE150 and COLO680 cells which were treated with IR 4Gy for 12 h (Additional file 1: Figure S1B). Nocodazole is a microtubule-disrupting agent to arrest cells in mitosis by triggering the mitotic checkpoint. Furthermore, we also determined cell cycle arrest induced by Nocodazole (0.5μg/ml) for 12 h. We demonstrated that, in KYSE150 cell line, G_2_/M ratio was diminished in these two mutant transfected cells after adding Nocodazole for 12 h. Meanwhile, the G1 phase enhanced relative to the wild-type cells. While in COLO680 cell line, the reducing ratio of G_2_/M in *TP53* R273H mutant transfected cells after adding Nocodazole treatment was not significantly statistic different, nevertheless, the decreasing ratio of G_2_/M in *TP53* G245C did have significantly difference, which suggest that G245C and R273H can abrogate cell response to Nocodazole induced cell cycle arrest (Additional file 1: Figure S1C). These results indicate that *TP53* G245C and R273H confer stronger resistance to UV- and IR- induced apoptosis and attenuate UV, IR as well as Nocodazole induced cell cycle arrest.

### The impacts of *TP53* R273H and R273C on the expression of P21, GADD45α and PARP

**Fig. 5:**
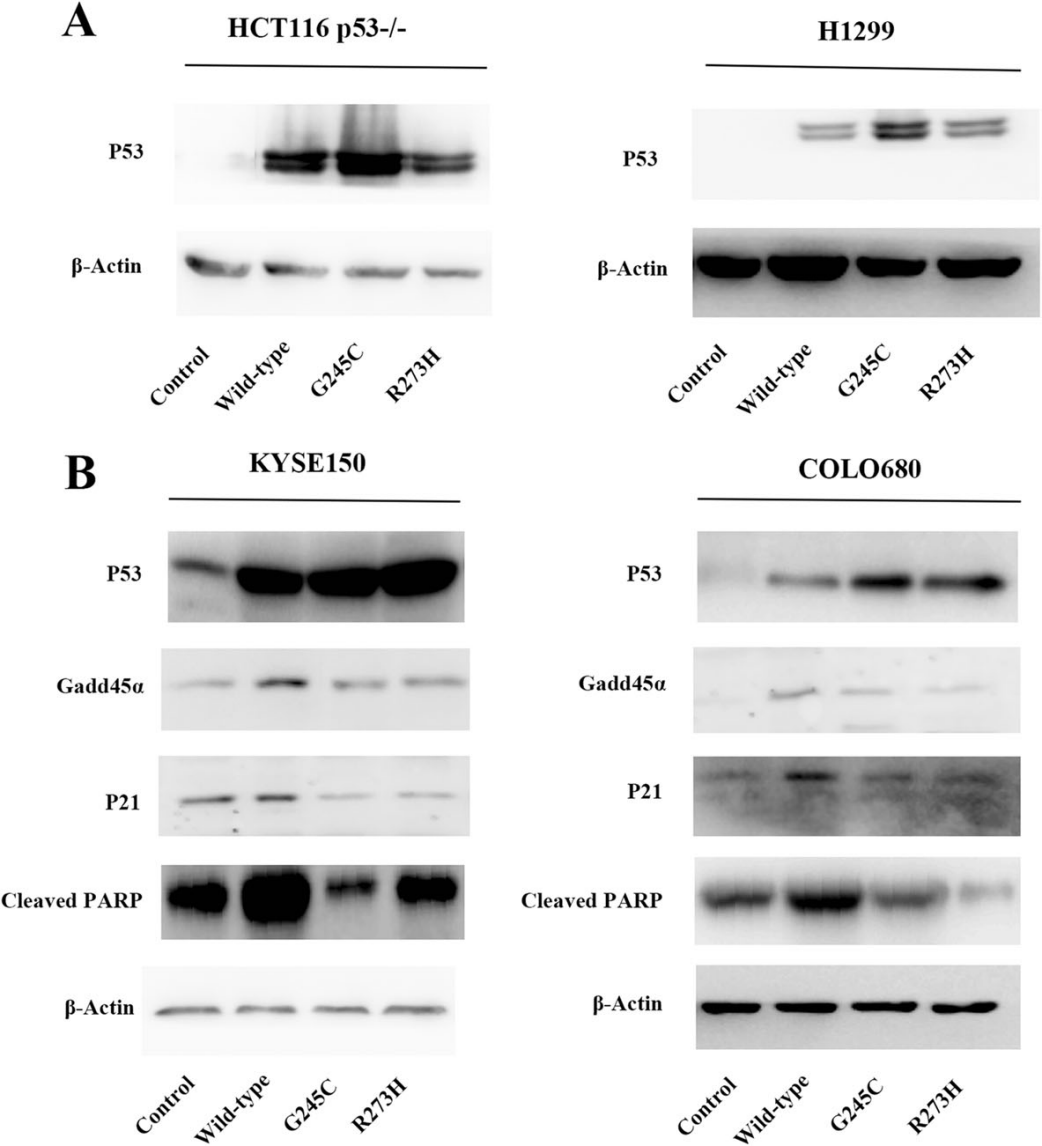
*TP53* and its downstream proteins’ expression levels. **a** The expression levels of P53 in p53-defective cell lines H1299 and HCT116 p53−/− after UV 30 J for 12 h treatment. **b** Mutant *TP53* R273H and R273C reduced the protein levels of P21, GADD45α and cleaved PARP after UV 30 J for 12 h treatment in KYSE150 and COLO680. Western blot of *TP53* downstream proteins P21, GADD45α and cleaved PARP expression levels in KYSE150 (right) COLO680 (left) cell lines after UV 30 J treatment for 12 h

Increasing evidences suggest that tumor cells express highly stabilized mutant *TP53* even in the absence of cellular stress, a prominent explanation is that mutant *TP53* fails to initiate the transcription of the MDM2, which consequently leads to a collapse of the negative feedback loop driven by MDM2 and the maintenance of high P53 levels in cells [17–19]. Additionally, the expressions of P53 were verified by Western blot in Fig. 5a. As shown in Fig. 5a, P53 expression levels were increased in *TP53* G245C and R273H mutant cells than wild-type and control cells.

*TP53* gene can be involved in many important cellular functions through the transcriptional regulation of its downstream genes. To further explore the mechanism of mutant *TP53* responses to cell cycle arrest and apoptosis, we examined the expression levels of several *TP53* downstream genes that participate in the process of cell cycle arrest and apoptosis, including *P21* and *GADD45α* which play vital roles in cell cycle arrest and apoptosis response, acting as both mediator and effector [20, 21]. Furthermore, the impacts of G245C and R273H mutations of *TP53* on the protein levels of P21 and GADD45α were determined, the two mutations were found greatly attenuated the induction of P21 and GADD45α after UV treatment (Fig. 5b). Hence, we speculate that G245C and R273H influence the process of cell cycle arrest and apoptosis might be due, at least in part, to down-regulate P21 and GADD45α protein levels. In addition, PARP is cleaved to PARP p85 rapidly during apoptosis [22]. Similarly, we have found that UV-induced cleaved PARP levels in control and WT *TP53* cells were elevated, but decreased markedly in G245C and R273H cells. These results further suggest that there exist a defect in UV-induced apoptosis in mutant cells (Fig. 5b).

## Discussion

*TP53* plays a vital important role in preventing tumor development. Under oncogenic or genotoxic stress, wild-type *TP53* is activated to inhibit cell growth through inducing cell-cycle arrest or apoptosis process [3]. It has been reported that more than 50% of human cancers carry somatic mutations of *TP53* [5] and that mutant *TP53* not only lose their tumor-suppressive functions [4, 8] but also acquire new transforming abilities to promote tumorigenesis, which are independent of wild-type *TP53* [9–12]. Consistent with previous reports, codons R273 and G245 are two of the most frequently mutant hot spots in the IARC database [23]. These two mutations have been proved to eliminate the function of wild-type *TP53* including *TP53* mediated cell cycle arrest and apoptosis, as well as to contribute to tumorigenesis through regulating some downstream genes. It is also evident that missense mutations may convert *TP53* from a tumor suppressor gene into an oncogene, and it is of both scientific and clinical interest to understand the gain of function activities of mutant *TP53* [11]. In this study, we identified these two mutations in clinical ESCC patients’ samples and investigated their oncogenic roles in ESCC cells. We found that *TP53* G245C and R273H could promote cell proliferation, colony formation, invasion and migration activities. Moreover, *TP5 3*G245C and R273H mutations confer stronger resistance to UV- and IR- induced apoptosis and attenuate UV, IR as well as Nocodazole induced cell cycle arrest.

Mutant P53 protein often accumulates at extremely high levels in tumors, which is partly due to the disruption of the transactivation of MDM2, which serves as a negative feedback loop down-regulating P53 protein levels in the absence of DNA damage signals and maintaining wild-type P53 at the low levels in normal tissues [17–19]. Wild-type P53 protein primarily functions as a transcription factor to transactivate many genes in the progress of cell cycle arrest and apoptosis, while mutant P53 protein abrogates transactivation of these target genes such as *P21, GADD45α* and *PARP* [1, 7]. Cyclin-dependent kinase (CDK) inhibitor P21 (also known as *P21*^*WAF1/Cip1*^), acts as an important transcriptional target of *TP53* [24] to inhibit the kinase activity of CDK4,6/cyclin-D and CDK2/cyclin-E leading to G_1_/S and G_2_/M cell cycle arrest in response to *TP53* activation after cellular or genomic stress [20, 25, 26]. *P21* is a tumor suppressor gene, but it can also serve as an oncogene in certain cellular process of human cancers [24]. In our results, we found that both *TP53* G245C and R273H mutations abolished wild-type p53 activated induction of P21 protein after UV treatment (Fig. 5b). A direct outcome of the scenario is shown by the *TP53* induced *P21* mediated G_1_/S arrest of these cancer cells, which is evacuated when the G245C and R273H mutations express high levels in the cells.

It has been repeatedly found that GADD45α is induced by DNA damage and other stress signals like UV, IR or Nocodazole, which are associated with cell cycle arrest and apoptosis [21, 27, 28]. GADD45α can displace PCNA from the cyclin D1 complex to prevent DNA replication during S phase [27], bind to *CDK1* to inhibit CDK1/cyclin B1 activity in vitro [21], and behave as a bridge between P53-dependent cell cycle checkpoint and DNA repair [28]. GADD45α is necessary for normal *TP53* activation by stress signals and its transcription can be stimulated by *TP53*. Its level rises notably in apoptotic cells, while inhibition of GADD45α expression level reduces apoptosis in response to DNA damage [28]. In our study, we have found that the induction of GADD45α expression was reduced dramatically after UV treatment in the cells expressing mutant *TP53* G245C and R273H, while it is up-regulated consistently in wild-type *TP53* cells (Fig. 5b), which suggests that these two *TP53* mutations lose the abilities to transcriptionally activate GADD45α and such reduction of GADD45α expression further attenuate apoptosis. Hence, knowing the particular *TP53* mutation of cancer may have utility for management in clinical practice since apoptosis is a major effect of chemotherapy or radiotherapy.

PARP enzyme plays an important role in DNA damage repair, differentiation, apoptosis, transcription, and DNA replication [29, 30]. PARP is converted from the 116-kDa form to fragments of 89 and 24 kDa and can be cleaved to PARP p89 early during the process of apoptosis [22, 31]. The cleaved PARP is one of biomarkers for the detection of apoptosis. In addition, PARP is a target of apoptotic caspase-3 and its two proteolytic fragments have been considered to be sign of functional caspase activation [32]. In the study, we have observed that UV treatment lead to increasing cleaved PARP levels in control and wild-type *TP53* cells, but decreasing in cells expressing G245C and R273H (Fig. 5b), which suggest that there exist a defect in UV-induced apoptosis in the mutant *TP53* cells.

Interestingly, we have identified *TP53* mutations (G245C and R273H) are distributed only in group of smokers in ESCC (5/88) and none is found in non-smokers group (Additional file 2: Figure S2) [16], which suggest that *TP53* mutations may be induced by tobacco exposure and lead to ESCC progress. As it is reported that several *TP53* point mutations are related to the effect of cigarette exposure in lung cancers [33–35] and squamous cell carcinomas of the head and neck [36], which is similar to our finding in patients with ESCC, a group that the percentage of smokers have higher *TP53* mutations. Undoubtedly, it is possible that the link between tobacco-induced *TP53* mutations and ESCC is influenced by other factors like tumor type and gender, age and ethnic of the patients [33], so realizing the relationship between tobacco-induced *TP53* mutations and ESCC will be a powerful tool that connect disease to its specific cause.

## Conclusions

In summary, this study has demonstrated that two malignant *TP53* mutations (G245C and R273H) revealed strong oncogenic properties in ESCC cells, indicating that such *TP53* mutations may play important roles in carcinogenesis and development of malignancy of ESCC. Given the important roles of mutant *TP53* G245C and R273H in tumorigenesis and enhancing cancer cell malignancies, it might have contributions to future diagnosis and therapy in *TP53* mutant cancers.

## Methods

### Plasmids construction

P53-SN3 was constructed from 1.8 KB-length of *TP53* cDNA sequence which was cloned into the *BamHI* sites of the expression vector. Two mutant *TP53* plasmids G245C and R273H were generated following the manuscript of QuikChange® Site-Directed Mutagenesis Kit (Agilent, USA).

### Cell culture and transiently transfection

The human ESCC cell lines (KYSE2, KYSE30, KYSE70, KYSE180, KYSE410, KYSE450, KYSE140, KYSE510 and COLO680) were provided by Professor Y. Shimada of Kyoto University. The HCT116 p53−/− cell line was brought from University of Pittsburgh by Professor Qimin Zhan. The H1299 cell line was purchased from ATCC and the catalogue is CRL-5803™. The ESCC cell lines KYSE2, KYSE30, KYSE70, KYSE140, KYSE180, KYSE410, KYSE450, KYSE510, COLO680 and the human lung cancer cell line, H1299 were cultured in 90% RPMI1640 medium, with 10% fetal bovine serum and antibiotics, at 37 °C, 5% CO_2_; KYSE150 was cultured in the medium of 1:1 mixture of Ham’s F12 and RPMI-1640 containing 2% FBS and antibiotics at humidified atmosphere with 37 °C, 5% CO_2_, the human colorectal cancer cell line, HCT116 p53−/− was grown in 90% DMEM medium with 10% fetal bovine serum, at 37 °C, 5% CO_2_. When cells were grown to 70–80% confluence, they were transiently cotransfected with the relative plasmids and siRNAs simultaneously using Lipofectamine^2000^ (Invitrogen) according to the standard procedure. For siRNAs transfection and plasmids transfection, the density of cells was grown to 40–50% and 70–80% individually.

### Cell proliferation assays

MTS assays were employed to analyze cell viability by a dye staining method using MTS reagent. 3000 cells per well were suspended in 96-well plates with 200 μl medium and grown for 96 h. Then, cells were incubated with MTS (0.5 mg/ml) and measured on an ELISA plate reader with the wavelength of absorbance at 490 nm and 630 nm at 0 h, 24 h, 72 h and 96 h of culture for proliferation assays, and percentage of respective basal levels (MTS concentrations of wells without cells) was calculated.

For a colony formation assay, cells were seeded in 60 mm plates at a concentration of 500 cells per well and changes culture with media every 72 h. Colonies were fix with methanol and stained with methanol and 0.5% crystal violet-acetic acid solution after 14 days, then colonies were visualized by fluorescence microscope and quantitated.

### Apoptosis and cell cycle detection assays

For apoptosis studies, an Annexin V-FITC/PI apoptosis assay kit (NeoBioscience, Shenzhen, China) was employed. After 48 h transfection, cells were washed with PBS, stained with Annexin V-FITC and propidium iodide (PI) according to the manufacturer’s protocol and quantitated by using flow cytometry (Becton-Dickinson, San Jose, CA, USA) to examine the apoptotic cell numbers.

Wild-type and mutant *TP53* transfected cells were seeded in six-well plate at a density of 10^6^ cells per well. After 48 h, cells were collected by trypsinization and centrifugation and washed with PBS. The cells were fix with precool 70% ethanol (*v*/v) overnight at − 20 °C, then centrifugation and washed with PBS. The cells were stained with PI (Propidium, 50μg/ml) 30 min at 37 °C and analyzed by flow cytometry (Becton-Dickinson, San Jose, CA, USA). Percentage of cell cycle cells was calculated according to the number of cells in the respective phase.

### Western blotting analysis

Cells were transfected with wild-type and mutant *TP53* plasmids for 48 h, total cell lysates were collected using lysis buffer (1 × PBS + 4%NP-40 + 0.2%proteinase inhibitor), 2 × SDS–PAGE sample loading buffer, and boiled for 5 min. 80 μg protein was performed and fractionated by SDS–PAGE, then semi-dry transferred to polyvinylidene difluoride membrane which was probed with antibodies for relative antibodies. Secondary antibodies were incubated, then visualized and quantified using Image Quant software (GE Healthcare Biosciences, Pittsburg, PA).

### Genomic DNA extraction and sequencing

Total genomic DNA was extracted in ten ESCC cell lines using TIANamp Genomic DNA kit (TIANGEN, Cat #DP304–03) according to the standard procedure. Each DNA (2 μl) was amplificated by PCR and 11 exons of *TP53* were sequenced (Sino Geno Max).

**Table:** 

exon	Forward	Reverse
exon1	GGAGCCTCGCAGGGGTTGATGG	CAAGTTCAGTCAGGAGCTTACC
exon2,3 and 4	CCTCTTGCAGCAGCCAGACTG	GCAACTGACCGTGCAAGTCA
exon5 and 6	GCTGCCGTGTTCCAGTTGCT	GCCACTGACAACCACCCTTA
exon7,8 and 9	TGCCACAGGTCTCCCCAAGG	CCCAAGACTTAGTACCTG
exon10 and 11	CCTCTGTTGCTGCAGATCCG	GTAGCCTGCACTGGCGTTCACC

### RNA extraction and quantitative real-time PCR (qRT-PCR)

Total RNA was isolated using Trizol reagent (Invitrogen) according to standard procedure. A reverse transcription reaction was performed using the one-step Reverse Transcription kit (Promega). Each cDNA (2 μg) was amplified in a SYBR Green Realtime PCR Master Mix (Thermo) and loaded on the Applied Biosystems 7300 Real-time PCR Detection System (ABI, Foster City, USA). GAPDH mRNA was employed as an endogenous control for mRNA. Thermal cycling conditions were as follows: the first one step, 95 °C for 30s and the ensuing 40 cycles, 95 °C for 5 s, 65 °C for 31 s, and melt curve step: 95 °C for 15 s, 65 °C for 1 min, 95 °C for 15 s. The relative expression levels of each group were quantified using the 2^-∆CT^ method and each mRNA level was normalized to the GAPDH mRNA. Primers used for qRT-RCR are listed as following:

**Table:** 

Gene	Forward	Reverse
*GAPDH*	TATGACAACAGCCTCAAGAT	AGTCCTTCCACGATACCA
*TP53*	CCACCATCCACTACAACTACAT	AGGACAGGCACAAACACG

### Invasion and migration assays

To prepare the invasion assay, a Matrigel™ invasion chamber (BD Biosciences, Bedford, MA) was employed to evaluate the invasive activity of *TP53* mutant and WT cells. Thoroughly mixing the sufficient Matrigel™ matrix with serum-free RPMI basal medium and carefully adding 0.1 ml of the diluted Matrigel matrix solution to each insert, then using the Matrigel™ matrix solution to incubate plates for 2 h in culture incubator, 37 °C, 5% CO_2_. Preparing cell suspensions in serum-free RPMI basal medium containing 3 × 10^4^ cells/ml for the 24-well chamber and subsequently filled with 600-800 μl RPMI-1640 medium with 20% FBS into each wells under the chambers. Cell invasion chambers were incubated in a humidified tissue culture incubator at 37 °C, 5% CO_2_. After overnight incubation, cells which invaded through the Matrigel™ membrane were fixed with methanol and stained with methanol and 0.5% crystal violet-acetic acid solution.

For the migration studies, the method as previously described and excluding the Matrigel™ matrix solution preparation was used. Invasive and migration cell numbers were visualized by fluorescence microscope and quantitated.

### Statistical analysis

Data for cell proliferation, invasion, migration, cell cycle and apoptosis were obtained at least in triplicate and employing GraphPad Prism v.5 software, expressed as mean ± SD (*n* ≥ 3). Quantization level of western blot was by Image J 1.8.0 software. Statistical differences were analyzed by the Student’s t-test (two-tailed), while analysis of variance (ANOVA) was used to determine whether there were any significant differences between groups. A *P*-value of less than 0.05 was considered to reflect significant differences. For ESCC clinical data, differences between groups were analyzed by the Fisher’s exact test.

## Additional files

Additional file 1: **Figure S1.** G245C and R273H reduced IR-induced apoptosis and weakened cell cycle arrest. (A) The portion of KYSE150 and COLO680 cells undergoing apoptosis was tested by flow cytometry induced by IR (4Gy) for 0 h and 12 h and the cells were dyed by PI and Annexin V. (B) Quantitation of cell cycle arrest ratio in WT and mutant *TP53* cells were treated with IR at 4Gy for 0 h and 12 h in KYSE150 and COLO680 cells. Results were shown as the percentage of DNA amounts dyed by PI which was detected by flow cytometry. Data was shown as mean ± SE (*P < 0.05). (C) Ratio of cell cycle arrest in WT and mutant *TP53* cells were treated with Nocodazole (0.5μg/ml) for 0 h and 12 h in KYSE150 and COLO680 cells. Data was shown as the quantitation of DNA amounts dyed by PI which was detected by flow cytometry. Data was shown as mean ± SE (*P < 0.05). (TIF 2588 kb)

Additional file 2: **Figure S2.** Clinical characteristics of the ESCC patients with *TP53* mutations (G245C and R273H) in smokers and non-smokers (Fisher’s exact test, *P* = 0.107). (TIF 8844 kb)

## Abbreviations

CDK: Cyclin-dependent kinase
DBD: DNA binding domain
ESCC: Esophageal Squamous Cell Carcinoma
GADD45α: Growth arrest and DNA-damage-inducible protein 45 alpha
GOF: Gain of function
IR: Ionizing radiation
LOF: Loss of function
PARP: Poly ADP-ribose polymerase
siRNAs: Short interfering RNAs
UV: Ultraviolet radiation
WGS: Whole genome sequencing
WT: Wild-type
