# Supplementary figures and images for: Mutant TP53 G245C and R273H promote cellular malignancy in esophageal squamous cell carcinoma
Source: BMC Cell Biol. 2018 Aug 20;19:16. doi: 10.1186/s12860-018-0167-y (PMC6102840; doi:10.1186/s12860-018-0167-y)

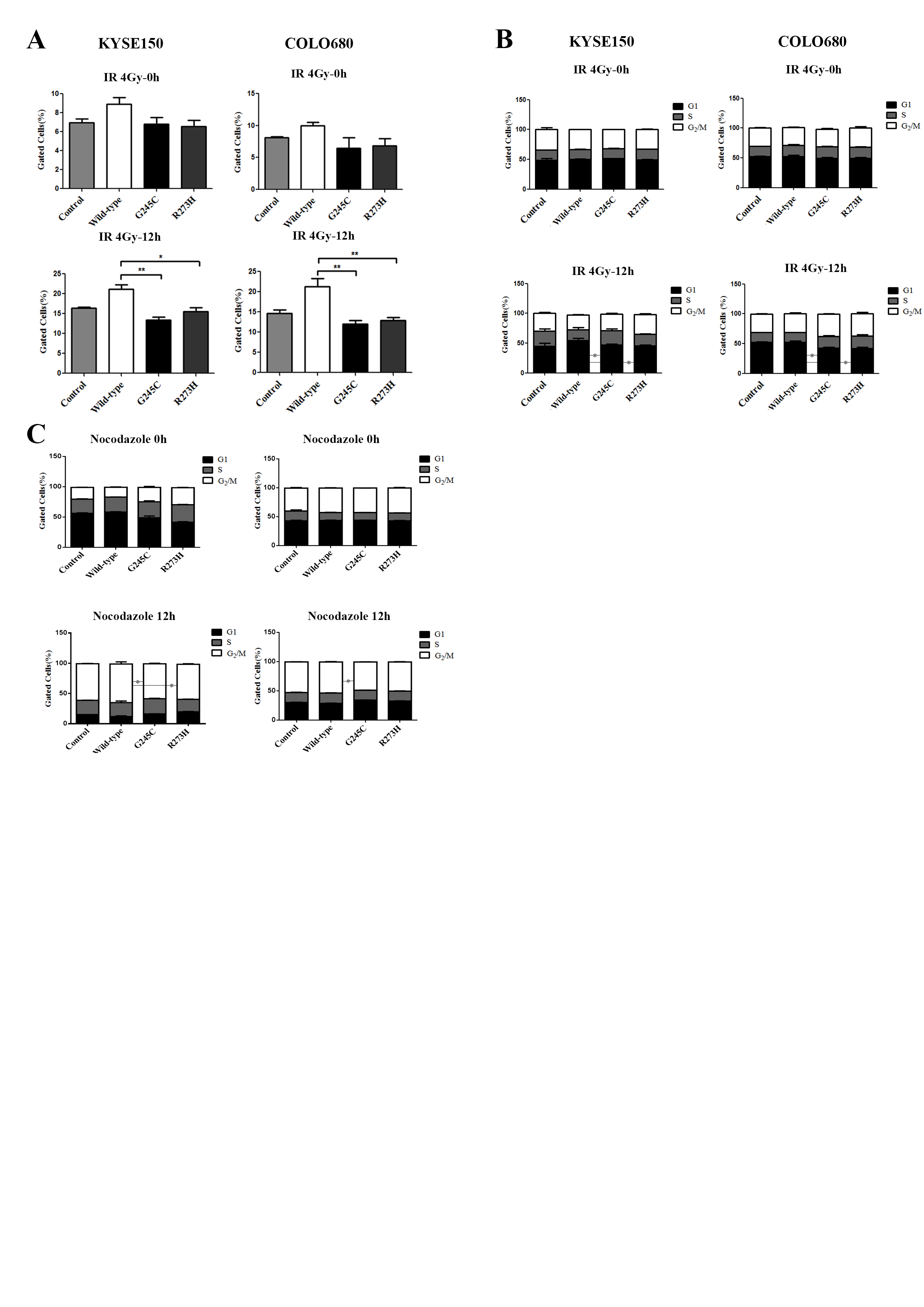

Supplement: Supplementary file 1 — Figure S1. G245C and R273H reduced IR-induced apoptosis and weakened cell cycle arrest. (A) The portion of KYSE150 and COLO680 cells undergoing apoptosis was tested by flow cytometry induced by IR (4Gy) for 0 h and 12 h and the cells were dyed by PI and Annexin V. (B) Quantitation of cell cycle arrest ratio in WT and mutant TP53 cells were treated with IR at 4Gy for 0 h and 12 h in KYSE150 and COLO680 cells. Results were shown as the percentage of DNA amounts dyed by PI which was detected by flow cytometry. Data was shown as mean ± SE (*P < 0.05). (C) Ratio of cell cycle arrest in WT and mutant TP53 cells were treated with Nocodazole (0.5μg/ml) for 0 h and 12 h in KYSE150 and COLO680 cells. Data was shown as the quantitation of DNA amounts dyed by PI which was detected by flow cytometry. Data was shown as mean ± SE (*P < 0.05). (TIF 2588 kb) [file 12860_2018_167_MOESM1_ESM.tif]

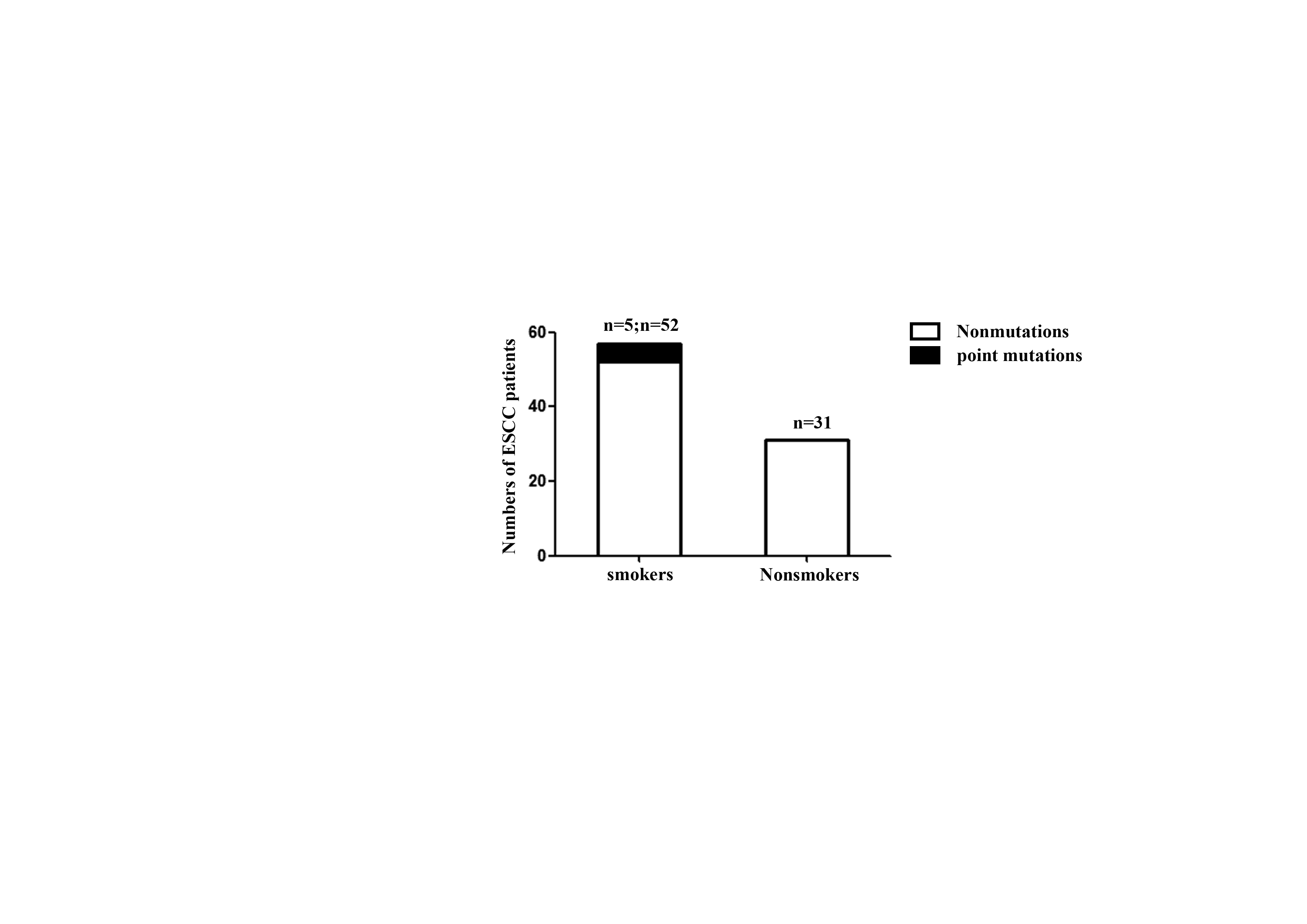

Supplement: Supplementary file 2 — Figure S2. Clinical characteristics of the ESCC patients with TP53 mutations (G245C and R273H) in smokers and non-smokers (Fisher’s exact test, P = 0.107). (TIF 8844 kb) [file 12860_2018_167_MOESM2_ESM.tif]
